# Early spot sign is associated with functional outcomes in primary intracerebral hemorrhage survivors

**DOI:** 10.1186/s12883-021-02146-3

**Published:** 2021-03-20

**Authors:** Wen-Che Tseng, Yu-Fen Wang, Tyng-Guey Wang, Ming-Yen Hsiao

**Affiliations:** 1 Department of Physical Medicine and Rehabilitation, National Taiwan University Hospital, 7, Zhongshan S. Rd., Taipei, Taiwan; 2 Department of Medical Imaging, National Taiwan University Hospital, 7, Zhongshan S. Rd., Taipei, Taiwan; 3 Department of Physical Medicine and Rehabilitation, College of Medicine, National Taiwan University, 7, Zhongshan S. Rd., Taipei, Taiwan

**Keywords:** Intracerebral hemorrhage, Spot sign, Functional outcome, Prognosis, Survivors

## Abstract

**Background:**

The computed tomography angiography (CTA) spot sign is a validated predictor of hematoma expansion and 30-day mortality in intracerebral hemorrhage (ICH). However, whether the spot sign predicts worse functional outcomes among ICH survivors remains unclear. This study investigated the frequency of the spot sign and its association with functional outcomes and length of hospital stay among ICH survivors.

**Methods:**

This was a retrospective analysis of consecutive patients with primary ICH who received CTA within 24 h from presentation to admission to the emergency department of a single medical center between January 2007 and December 2017. Patients who died before discharge and those referred from other hospitals were excluded. CTAs with motion artifacts were excluded from the analysis. The presence of a spot sign was examined by an experienced neuroradiologist. Functional outcomes were determined based on the modified Rankin Scale (mRS) score and Barthel Index (BI). Severe dependency in activities of daily living (ADL) was defined as BI of ≤60 and severe disability as an mRS score of ≥4. Odds ratio (OR) and multiple linear regression were used as measures of association.

**Results:**

In total, 66 patients met the inclusion criteria, of whom 9 (13.64%) were positive for a spot sign. No significant differences were observed in baseline characteristics between patients with and without a spot sign. Patients with a spot sign tended to be severely dependent in ADL at discharge (66.67% vs 41.07%; OR = 2.87; *p* = 0.15) and were more likely to require ICH-related surgery (66.67% vs 24.56%; OR = 6.14; *p* = 0.01). In multiple linear regression, patients with a higher spot sign score had a significantly longer hospital stay (coefficient = 9.57; 95% CI = 2.11–17.03; *p* = 0.013).

**Conclusions:**

The presence of a spot sign is a common finding and is associated with longer hospital stay and possibly worse functional outcomes in ICH survivors.

## Background

Primary intracerebral hemorrhage (ICH) is the second most common type of stroke, accounting for 10–30% of all cases [1]. The incidence rate of primary ICH was estimated to be 22 per 100,000 person-year in low- to middle-income countries and 10 per 100,000 person-year in high-income countries between 1970 and 2008 [1]. Globally, between 1990 and 2013, the age-adjusted prevalence of hemorrhagic stroke remained stationary, but the age-adjusted mortality of hemorrhagic stroke decreased [2], resulting in an enhanced survival rate. However, many ICH survivors have neurological and functional impairment, requiring long-term inpatient or outpatient rehabilitation. In 2015, a population-based study revealed that only 14% of the patients with primary ICH achieved functional independence, defined as a modified Rankin Scale (mRS) score of ≤2, at 1 year [3].

The computed tomography angiography (CTA) spot sign was first described in 1999 when the investigators found that the extravasation of radiographic contrast was an independent predictor of mortality in patients with primary ICH [4]. Strict radiological criteria and scoring system have been developed to better identify and quantify the spot sign [5]. In 2019, a meta-analysis revealed a high area under the receiver operating characteristic curve for ICH growth and mortality (0.86 and 0.87, respectively), indicating good sensitivity and specificity of the spot sign to predict these outcomes [6].

Since its first report, many studies have attempted to establish the outcome prediction value of the CTA spot sign. Several studies have demonstrated the association of the CTA spot sign with higher mortality rates [7–9] and the predictive value of the spot sign in hematoma expansion [10, 11] in patients with primary ICH. The CTA spot sign is also associated with higher mRS scores at 3 months [7–9], and it serves as a predictor of re-bleeding after endoscopic surgery for ICH [12]. In addition, the spot sign may serve as a marker for potential candidates of early hemostatic therapy after ICH. Several trials using tranexamic acid and recombinant activated factor VII are ongoing or have been recently completed [13, 14].

Despite these insights, earlier studies on the CTA spot sign and functional outcomes are limited. These studies used mRS as the only measure of functional outcome, and the calculation of mRS included patients who died (mRS = 6) in the acute phase of the disease [9, 15]. Additional evaluation of the association between the CTA spot sign and activities of daily living (ADL) functions in ICH survivors is warranted. Furthermore, the predictive value of the CTA spot sign for other ICH-related outcomes such as length of hospital stay or requirement of surgical intervention remains unestablished. This has an important clinical implication: the CTA spot sign can serve to identify ICH survivors with worse ADL functions at discharge, potentially experiencing long-term disabilities, and who are primary candidates for post-acute rehabilitation.

In the present study, we investigated the frequency of the CTA spot sign and its association with outcomes at hospital discharge, focusing primarily on independency in ADL, among ICH survivors.

## Methods

### Patients

We performed a retrospective analysis of consecutive patients with ICH admitted to the rehabilitation, neurology, or neurosurgery ward who were receiving CTA within 24 h from presentation to admission to the emergency department of a single medical center between January 2007 and December 2017. Patients who died before discharge, were younger than 20 years, and were referred from other hospitals were excluded. CTA with motion artifacts was also excluded from the analysis. Patients with etiologies other than primary ICH (e.g., aneurysm rupture, arteriovenous malformation, and tumor bleeding) were excluded.

### Image acquisition

The scanning protocol included a pre-contrast scan and multiphase CT Angiography. CT angiography was performed by using multiple scanners, including 64-, 128-, and 320-section machines (General Electric Medical Systems and Siemens). Timing-bolus technique was used for optimal enhancement. The CT angiography injection protocol included 60–75 mL of contrast medium (Ultravist 370 [iopromide; Bayer, Leverkusen, Germany] or Omnipaque 350 [iohexol; GE Healthcare, Chicago, Illinois]) injected for 15 s at a flow rate of 4–5 mL/s, followed by a 20-mL saline solution chaser through a 20-gauge venous catheter. A 3-phase CT angiogram was acquired, with a 2-s interval between scans. The first phase covered the area from the aortic arch to the vertex, and the subsequent 2 phases covered the cranium only. The delayed-phase CTA images were acquired to survey delayed spot signs as well as to exclude dural venous sinus thrombosis, which is also a common cause of intracerebral hemorrhage, or other spot sign mimics such as tiny aneurysms. The CT angiographic image was reformatted into 0.625-mm axial and 1-mm sagittal and coronal images. Delayed-phase 0.625-mm axial images were also available.

### Outcome measures

The presence of a spot sign was retrospectively examined by an experienced neuroradiologist, who was blinded to patient data, and the spot sign score Appendix, Table 5) was calculated. Functional outcomes were determined based on the mRS score and Barthel Index (BI) at discharge (Appendix, Table 3 and 4). Severe dependency in ADL was defined as BI of ≤60 and severe disability was defined as an mRS score of ≥4. Functional independence was defined as a BI of > 90 or an mRS score of ≤2. All mRS, BI, and ADL were calculated by the primary care physicians. The number of patients who received related brain surgery or ventriculostomy and the length of hospitalization were also analyzed.

### Statistical analyses

The data was analyzed with Stata Statistical Software 14.0 (StataCorp LP., College Station, Texus, USA). Patient demographics and baseline characteristics were analyzed using descriptive statistics and are presented as mean ± SD or percentage. Pearson’s chi-squared test or the Fisher’s exact test was used to examine the association between categorical variables, and the *t*-test was used to examine the association between numerical variables and the spot sign. Multiple linear regression was used to assess the association between the spot sign and clinical outcomes. A *p* value of < 0.05 was considered statistically significant.

## Results

**Fig. 1:**
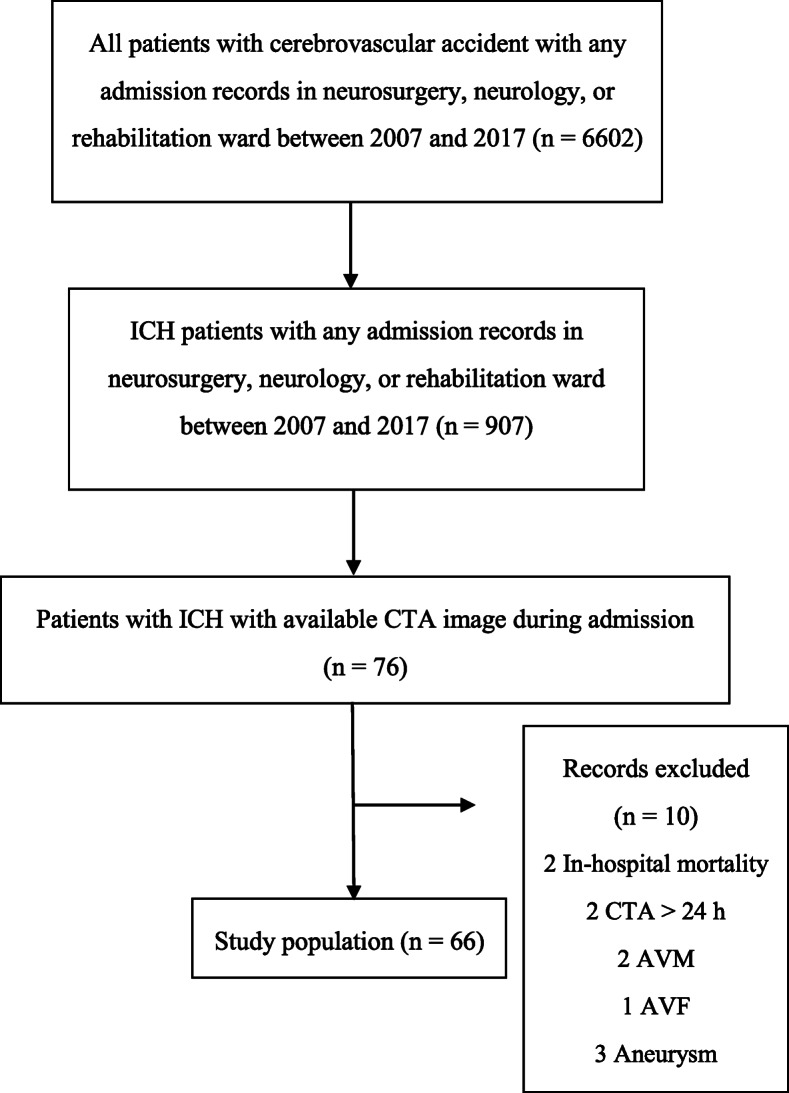
Diagram of patient selection

A total of 6602 patient records and brain images were reviewed, and 66 patients met the inclusion criteria (Fig. 1).

Among primary ICH survivors, 9 (13.64%) had a spot sign (Table 1). On average, BI improved from a mean of 19.17 to 62.77 during a mean of 42.15 days of hospitalization. Less than half (29; 44.62%) of the patients were severely dependent in ADL at discharge, and only 10 patients (15.15%) achieved functional independence, as assessed by BI ≤60 or > 90, respectively.

**Table 1:** Baseline characteristics of the population (*n* = 66)

Variables	*N (% or SD)*
Age (years)	55.56 (14.85)
Gender	
Female	24 (36.36)
Male	42 (63.64)
GCS at admission	
3–8	12 (18.18)
9–13	18 (27.27)
14–15	36 (54.55)
Presence of spot sign	9 (13.64)
Spot sign score	
1	3 (33.33)
2	3 (33.33)
3	2 (22.22)
4	1 (11.11)
BI at admission	19.17 (20.56)
BI at discharge	62.77 (25.77)
Severe dependency	
BI ≤ 60	29/65 (44.62)
mRS ≥ 4	25 (37.88)
Functional independence	
mRS ≤ 2	7/66 (10.61)
BI > 90	10/66 (15.15)
Length of hospitalization (days)	42.15 (25.37)

*N* Number, *SD* Standard deviation, *GCS* Glasgow Coma Scale, *BI* Barthel Index, *mRS* modified Rankin Scale

**Fig. 2:**
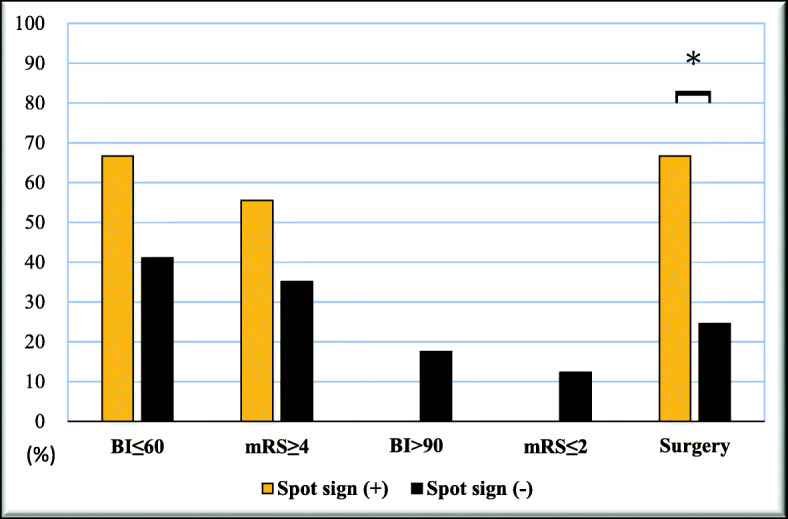
Functional outcomes of patients with or without a spot sign. ^*^ *p* < 0.05

No significant differences were observed in age, sex, and BI and Glasgow coma scale (GCS) score at admission between patients with and without a spot sign (Table 2). Regarding functional status, patients with a spot sign tended to be severely dependent in ADL (BI ≤60) at discharge (66.67% vs 41.07%; odds ratio [OR] = 2.87; *p* = 0.15) and were more likely to have profound disability (mRS score ≥ 4) (55.56% vs 35.09%; OR = 2.31; *p* = 0.24; Fig. 2). All the patients who achieved functional independence (*n* = 10, defined by BI > 90; or *n* = 7 defined by mRS score ≤ 2) were negative for the spot sign (Fig. 2).

**Table 2:** Comparison between ICH survivors with or without a spot sign (*n* = 66)

Variables	Spot sign negative (*n* = 57) *N (% or SD)*	Spot sign positive (*n* = 9) *N (% or SD)*	*P*-value	*Odds Ratio (OR)*
Age (years)	55.26 (15.34)	57.44 (11.86)	0.69	
Gender (female)	20/57 (35.09)	4/9 (44.44)	0.59	
GCS at admission			0.85	
3–8	11 (19.30)	1 (11.11)		
9–13	14 (24.56)	4 (44.44)		
14–15	32 (56.14)	4 (44.44)		
BI at admission	19.39 (18.15)	17.78 (33.55)	0.41	
BI at discharge (mean)	63.39 (27.05)	58.89 (16.16)	0.32	
Severe dependency				
BI ≤ 60	23/56 (41.07)	6/9 (66.67)	0.15	2.8 ^a^
mRS ≥ 4	20/56 (35.09)	5/9 (55.56)	0.24	2.31^a^
Functional independence				
BI> 90	10/57 (17.54)	0/9 (0)	0.17	–
mRS ≤ 2	7/57 (12.28)	0/9 (0)	0.34	–
Surgery	14/57 (24.56)	6/9 (66.67)	0.01	6.14
Ventriculostomy	3/57 (5.26)	2/9 (22.22)	0.07	5.14
Length of hospitalization (days)	39.81 (24.00)	55.7 (30.20)	0.06	
Hematoma expansion	8/39 (20.51)	3/5 (60)	0.055	

*N* Number, *SD* Standard deviation, *GCS* Glasgow Coma Scale, *BI* Barthel Index, *mRS* modified Rankin Scale

^a^Odds ratio for worse outcome (BI ≤60 or mRS score ≥ 4)

In addition, two-thirds of the patients positive for a spot sign received surgery compared with only one-fourth of those negative for a spot sign (66.67% vs 24.56%; OR = 6.14; *p* = 0.01). In multiple linear regression adjusted for age and sex, a higher spot sign score was significantly associated with a longer hospital stay (coefficient = 9.57; 95% CI = 2.11–17.03; *p* = 0.013).

## Discussion

### Spot sign and functional outcomes

This study is the first to focus on the association of the spot sign and functional outcomes in primary ICH survivors. Our results demonstrated that patients with a spot sign tended to be severely dependent in ADL at discharge and were more likely to require surgical intervention. In addition, patients with a higher spot sign score had a significantly longer hospital stay. ICH survivors, whose numbers are steadily increasing over the decades, are candidates for long-term neuro-rehabilitation, and analyses focusing on this group have important clinical implications. Detection of a spot sign may have great benefit for designing personalized and intense rehabilitation programs for ICH survivors.

Our results demonstrated that in primary ICH survivors, the presence of a spot sign within 24 h of ICH onset may be associated with detrimental functional status at discharge. Although the data did not reach statistical significance, notably, none of the patients positive for a spot sign achieved functional independence. Earlier studies have revealed that the spot sign is associated with higher mortality, hematoma expansion, and worse functional outcomes in patients with ICH, indicating the predictive value of the spot sign. However, these studies determined the endpoint of functional outcome solely based on the mRS score and the study population included patients who died (mRS = 6) during the acute disease phase, which might obscure the predictability of the spot sign in ICH survivors [9, 15]. In the present study, we determined functional outcomes based on both BI and mRS scores, which are better in characterizing ADLs. Both parameters revealed that patients with a spot sign tended to have worse outcomes (BI: OR = 2.87, *p* = 0.15; mRS: OR = 2.31; *p* = 0.24). The OR for worse outcome defined based on the mRS score (≥4) was similar to previous studies, which reported ORs of 2.40 and 2.50 [7, 9]. The nonsignificance of the OR in the present study could be attributed to the small sample size, due to strict inclusion/exclusion criteria, and the selection of patients admitted to rehabilitation, neurology, and neurosurgery wards to retrieve consistent functional evaluation records.

### Spot sign, surgery, and length of hospital stay

Essentially, two-thirds of the patients positive for a spot sign required cranial surgery compared with only one-fourth of the patients negative for a spot sign (66.67% vs 24.56%, OR = 6.14; *p* = 0.01). Indication for surgery is often an ominous sign of the profound mass effect of hematoma, although numerous factors, such as location of hematoma and baseline condition of patients, may confound the decision. On the contrary, although the benefit of surgery in terms of mortality and functional outcomes remains unclear in primary ICH [16], recent studies have reported that early neurosurgery is predictive of longer survival and better functional outcomes in patients with severe primary ICH [17]. Thus, early identification of patients with ICH in whom surgical intervention has a positive impact is of great clinical importance.

In addition to functional outcomes, multiple linear regression demonstrated that patients with a higher spot sign score had a longer mean hospital stay (*p* = 0.013). Essentially, all the patients in this study were covered by the National Health Insurance program, which covers 99.9% of the citizens of Taiwan, attenuating the influence of private health insurance and socioeconomic status on the length of hospital stay. The duration of hospitalization may reflect the severity of diseases, complications rates, and medical costs. Furthermore, studies in geriatric and general populations indicate that prolonged hospitalization is associated with increased functional dependence, infection, and comorbidities [18–20].

### Implications

Early CTA serves as an important tool to diagnose and evaluate ICH, and an earlier study reported that a CTA spot sign could be precisely detected across multiple medical centers [21]. Extending its association with functional outcomes, as is shown in our results, the spot sign is a potential indicator for prognosis in patients with primary ICH. Furthermore, the spot sign helps identify ICH survivors who might require surgical intervention and longer in-patient treatment course. Combining the CTA spot sign with other prognostic tools is also an option [15], but more research on this topic is warranted.

### Limitations

This study has several limitations. First, the sample size was relatively small. CTA was not routinely performed in patients with ICH. Limited case number hinders further stratification according to BI and mRS scores. Second, the follow-up period was short and not standardized in all the patients because of the retrospective study design. Further large-scale prospective studies with longer follow-up durations are warranted to comprehensively verify the predictive value of the spot sign. Third, in our retrospective review, we found no clear protocol for when and on whom to perform CTA in patients with ICH. CTA was performed when the cerebral hemorrhage seemed atypical for primary hypertensive ICH, which was determined by neurologists or neurosurgeons. This could have caused a selection bias that not all patients with primary ICH have CTA data available, and the criteria for prescribing a CTA may differ between physicians. Despite these limitations, our study still provides important evidence of the association of an early spot sign and deleterious outcomes in primary ICH survivors.

## Conclusions

The presence of a spot sign is a common finding and is associated with longer hospital stay and possibly worse functional outcomes in ICH survivors. This study provides important evidence of the association of an early CTA spot sign and deleterious outcomes in primary ICH survivors.

## Data Availability

The datasets used and/or analysed during the current study are available from the corresponding author on reasonable request.

## Appendix

**Table 3:** Barthel index

Activity	Score
Feeding	0 = unable
	5 = needs help cutting, spreading butter, etc., or requires modified diet
	10 = independent
Bathing	0 = dependent
	5 = independent (or in shower)
Grooming	0 = needs to help with personal care
	5 = independent face/hair/teeth/shaving (implements provided)
Dressing	0 = dependent
	5 = needs help but can do about half unaided
	10 = independent (including buttons, zips, laces, etc.)
Bowels	0 = incontinent (or needs to be given enemas)
	5 = occasional accident
	10 = continent
_Bladder_	0 = incontinent, or catheterized and unable to manage alone
	5 = occasional accident
	10 = continent
Toilet	0 = dependent
	5 = needs some help, but can do something alone
	10 = independent (on and off, dressing, wiping)
Transfer	0 = unable, no sitting balance
	5 = major help (one or two people, physical), can sit
	10 = minor help (verbal or physical)
	15 = independent
Ambulation	0 = immobile or < 50 yards
	5 = wheelchair independent, including corners, > 50 yards
	10 = walks with help of one person (verbal or physical) > 50 yards
	15 = independent (but may use any aid; for example, stick) > 50 yards
Stairs	0 = unable
	5 = needs help (verbal, physical, carrying aid)
	10 = independent

**Table 4:** Modified Rankin scale

Score	Definition
0	No symptoms
1	No significant disability. Able to carry out all usual activities, despite some symptoms.
2	Slight disability. Able to look after own affairs without assistance, but unable to carry out all previous activities.
3	Moderate disability. Requires some help, but able to walk unassisted.
4	Moderately severe disability. Unable to attend to own bodily needs without assistance, and unable to walk unassisted.
5	Severe disability. Requires constant nursing care and attention, bedridden, incontinent.
6	Dead.

**Table 5:** Spot sign score

Definition	Point
**Number of spot signs**	
1–2	1
≥ 3	2
**Maximum axial dimension**	
1–4 mm	0
≥ 5 mm	1
**Maximum attenuation**	
120–179 HU	0
≥ 180 HU	1

## Abbreviations

ADL: Activities of daily living
BI: Barthel Index
CTA: Computed tomography angiography
GCS: Glasgow coma scale
ICH: Intracerebral hemorrhage
mRS: Modified Rankin Scale
OR: Odds ratio
